# CRISPR/Cas9 Knockout Studies Implicate Phenazine-1-carboxylic Acid, but Not 2-Hydroxy Phenazine, in the Biocontrol Activity of *Pseudomonas chlororaphis* Subsp. *phenazini* Strain S1Bt23 Against *Pythium arrhenomanes* (Drechsler)

**DOI:** 10.3390/microorganisms14010019

**Published:** 2025-12-20

**Authors:** Mercy Akuma, Sylvia Ighem Chi, Renlin Xu, Indira Thapa, Aida Kebede, Barbara Blackwell, James Tabi Tambong

**Affiliations:** 1 Ottawa Research and Development Centre, Agriculture and Agri-Food Canada, Ottawa, ON K1A 0C6, Canada; makum086@uottawa.ca (M.A.); sylvia.ighemchi@blood.ca (S.I.C.); renlin.xu@agr.gc.ca (R.X.); indira.thapa@agr.gc.ca (I.T.); aida.kebede@agr.gc.ca (A.K.); barbara.blackwell@agr.gc.ca (B.B.); 2 Department of Cellular and Molecular Medicine, University of Ottawa, Ottawa, ON K1N 8M5, Canada; 3 Canadian Blood Service, Ottawa, ON K1Z 7M3, Canada

**Keywords:** pyrrolnitrin, phenazine-2-ol, biological control, cell toxicity, liquid chromatography, thin-layer chromatography, oomycetes

## Abstract

Liquid chromatography–mass spectrometry analysis of *Pseudomonas chlororaphis* subsp. *phenazini* S1Bt23 extracts detected phenazine-1-carboxylic acid (PCA) and 2-hydroxyphenazine (2-OH-PHZ) as the main phenazine derivatives. We investigated their relative contributions to the antagonistic activity of strain S1Bt23 against *Pythium arrhenomanes*, a root rot pathogen of corn. CRISPR-Cas9 knockouts were carried out on the *phz*F gene, required for PCA synthesis, and the *phz*O gene, which is involved in converting PCA to 2-OH-PHZ. Deletion of the *phz*F gene abrogated the production of PCA and 2-OH-PHZ, and the Δ*phz*F mutant lost the antagonism against *Pythium arrhenomanes*. In contrast, deletion of the *phz*O gene created a 2-OH-PHZ-negative mutant with intact antagonistic ability. Concordantly, S1Bt23 wild type and the Δ*phz*O mutant, but not the Δ*phz*F mutant, significantly bioprotected corn seeds of a susceptible inbred variety, CO441, from *P. arrhenomanes*. At equimolar amounts of 75 nM, synthetic PCA inhibited *Pythium* growth, whereas 2-OH-PHZ did not. This highlights the critical contribution of PCA to the biocontrol activity of strain S1Bt23 against *P. arrhenomanes*. Unexpectedly, deletion of *phz*O did not result in additional PCA accumulation. This suggests that the conversion of PCA to 2-OH-PHZ by S1Bt23 is a potential protective mechanism against the overproduction of lethal cellular doses. This study paves the way for bioengineering strain S1Bt23 into a more effective biopesticide.

## 1. Introduction

Secondary metabolites produced by bacteria, especially pseudomonads, have been studied extensively due to their broad-spectrum antimicrobial activities and roles in virulence [1,2,3,4]. Among these, researchers have long focused on phenazines and their derivatives, as they are believed to be major contributors to antimicrobial activity. Phenazines constitute a large group of bright-colored nitrogen-containing heterocyclic compounds with diverse biological activities [5,6,7]. Over 100 different phenazine structural derivatives have been identified in nature, and even more have been derived synthetically [8]. In *Pseudomonas* spp., the highly conserved biosynthetic cluster *phzABCDEFG* is responsible for the conversion of chorismate, the end product of the shikimate pathway, into phenazine-1-carboxylic acid, which is the base substrate for the synthesis of phenazine derivatives [9,10,11]. In many phenazine-producing bacteria, this biosynthetic cluster is flanked by accessory genes encoding phenazine-modifying enzymes that produce strain-specific phenazines. For example, *Pseudomonas chlororaphis* subsp. *phenazini* strains (S1Bt23 and 30–84, previously *P. chlororaphis* 30–84). encodes *phz*O, a monooxygenase that converts a proportion of yellow phenazine-1-carboxylic acid (PCA) into orange 2-hydroxyphenazine-1-carboxylic acid (2-OH-PCA) [12,13]. Then, 2-OH-PCA is spontaneously decarboxylated to form 2-hydroxyphenazine (2-OH-PHZ). *P. chlororaphis* PCL1391 and *P. aeruginosa* PAO1 encode *phz*H, a transamidase that converts precursor PCA into phenazine-1-carboxamide (PCN) [14]. *phz*M, a methyltransferase [15], and *phz*S, a flavin-containing monooxygenase found in *P. aeruginosa* PAO1, together convert PCA into pyocyanin (PYO) [16,17]. However, PhzS, when expressed alone, can synthesize 1-hydroxyphenazine (1-OH-PHZ) from PCA [18]. It is therefore clear that the activities of one or a few terminal modifying enzymes are responsible for the diverse phenazine derivatives observed in nature. Given that these structural variations account for much of the diversity in the activity of phenazines, the presence of phenazine-modifying enzymes has a major impact on the ecological fitness and activity of the target strains.

Phenazine and its derivatives have diverse functions in virulence [19,20,21], regulation of gene expression [22], neuroprotection [23], and insecticidal, antimicrobial [24,25,26], antiparasitic [27], and antitumor activities [28]. Phenazine derivatives vary in the extent of their antimicrobial activities depending on the pathogen they are acting against. In agricultural applications, phenazines produced by fluorescent pseudomonads are associated with their ability to suppress various plant pathogens, such as fungi and nematodes [8,14,26,29,30]. For example, PCA, PCN, and hydroxyphenazines produced by *P. aeruginosa* inhibited the growth of the fungus *Aspergillus fumigatus* [31]. Similarly, PCA produced by *P. chlororaphis* subsp. *aureofaciens* strain M71 was found to play a critical role in its antagonistic activity against various plant pathogenic fungi [32]. Analysis of *P. chlororaphis* strain PA23 revealed that the antibiotic pyrrolnitrin (PRN) is more effective than PCA and 2-OH-PHZ against the fungal pathogen *Sclerotinia sclerotiorum* [33]. In the case of *P. fluorescens* strain Psd, which produces both PCA and PRN, PCA was determined to be more potent than PRN in the biocontrol of fungal phytopathogen *Fusarium oxysporum* [34]. One study showed that PCA produced by *P. chlororaphis* subsp. *aureofaciens* strain M71 showed more activity compared to 2-OH-PHZ against several plant pathogenic fungi [32]. In contrast, another study showed that 2-OH-PCA exhibits stronger fungistatic and bacteriostatic activity than PCA against the fungal pathogen *Gaeumannomyces graminis var. tritici* [35]. While the production of phenazines enhances the competitiveness and fitness of the producing strain, overproduction of these metabolites can also be toxic. One study investigated the survival of phenazine-deficient *P. aeruginosa* cells incubated in the presence of either PYO, PCA, 1-OH-PHZ, or PCN, which it typically produces [36]. Their results indicated that each phenazine derivative was toxic to different extents. Due to these properties, various phenazine-producing strains have emerged as useful biological control agents and are frequently used as active ingredients in biopesticides.

In this study, we further characterized novel *P. chlororaphis* subsp. *phenazini* S1Bt23, which displayed potent antagonistic activity against the oomycete *Pythium ultimum* [12]. The main objective of this study was to determine the key phenazine derivative(s) involved in the antagonistic activity of *P. chlororaphis* subsp. *phenazini* S1Bt23 against *P. arrhenomanes* (Drechsler) (PYTHAR), the root and stem rot pathogen of corn. S1Bt23 encodes *phz*O and was shown by liquid chromatography–mass spectrometry (LC-MS) analysis to primarily produce phenazine derivatives PCA and 2-OH-PHZ. Using CRISPR-targeted gene deletions, we demonstrated that the biocontrol of the maize root rot pathogen, *P. arrhenomanes* (Drechsler), by *P. chlororaphis* subsp. *phenazini* S1Bt23 is primarily due to PCA biosynthesis, not 2-OH-PHZ. Since the genome sequence of *P. chlororaphis* subsp. *phenazini* S1Bt23 has an intact pyrrolnitrin biosynthetic cluster, we also investigated its potential contribution and found it plays a minor role in the in vitro antagonistic ability of *P. chlororaphis* subsp. *phenazini* S1Bt23 against *P. arrhenomanes* (PYTHAR). *P. arrhenomanes* causes pre- and post-emergence corn seedling death [37,38], with 60% incidence recorded in some areas in Mexico [39]. *Pythium*-induced seedling blights and root rots lead to significant economic losses of almost USD 25 million to corn (*Zea mays*) production in Ontario (Canada) and the United States. Chemical fungicides such as etridiazole and metalaxyl are the primary control measures of *Pythium* diseases [40,41]. This strategy has negative impacts on public health and the environment, leading to significant biodiversity loss [42,43]. Biological control of *Pythium* is an emerging alternative and sustainable strategy.

## 2. Materials and Methods

### 2.1. Microbial Strains

The isolation of *P. chlororaphis* subsp. *phenazini* S1Bt23 is described in Tchagang et al. [44]. All bacterial strains were grown in Luria–Bertani (LB) (Sigma, Oakville, ON, Canada), unless otherwise indicated, at 28–30 °C for strain S1Bt23 and its mutants, while *Escherichia coli* was cultured at 37 °C. Antibiotic concentrations used were 50 µg/mL kanamycin (S1Bt23 and *E. coli*) and tetracycline at 100 µg/mL or 20 µg/mL for *P. chlororaphis* subsp. *phenazini* S1Bt23 or *E. coli*, respectively. *P. arrhenomanes* isolate LEV1578 (PYTHAR) was cultivated on potato dextrose agar (PDA; BD Difco™, Mississauga, ON, Canada) at 28 °C.

### 2.2. In Vitro Dual Culture and Corn Seed Germination Assays

Dual culture assays were used to test the antagonistic activity of wild-type and mutant S1Bt23 strains against the oomycete *P. arrhenomanes* as previously described [44]. Briefly, a plug (~5 mm in diameter) of the oomycete culture was transferred from a 3-day-old agar plate to the center of a glucose–casamino acid–yeast agar (GCY; glucose 15 g/L, casamino acids 1.5 g/L, yeast extract 1.0 g/L, KH_2_PO_4_ 1.5 g/L, MgSO_4_∙7H_2_O 1.0 g/L, agar 15 g/L). Bacteria were streaked at equidistance on both sides of the oomycete plug, incubated at 30 °C, and monitored daily. Five replicates were streaked per bacterial strain. Inhibition rate (%) was calculated using the formula ([G_f_ − G_f+b_]/G_f_) × 100, where G_f_ is the radial growth distance of the oomycete alone, and G_f+b_ is the radial growth distance towards the streaked bacteria. For biocontrol assays involving extracts and synthetic compounds, a plug of *P. arrhenomanes* (PYTHAR) culture was transferred from a 3-day-old PDA plate to the center of a GCY agar plate. Twenty microliters of corresponding extracts or compounds were then spotted at the indicated locations. Five replicate plates of each bacterial treatment were incubated at 30 °C and monitored daily for the inhibition zone.

Two hundred and fifty corn (*Zea mays*) seeds (inbred CO441) were disinfected for 15 min in 5% Clorox amended with 2 drops of Tween 80 with shaking (250 rpm). The seeds were then rinsed 4 times in sterilized deionized water (diH_2_O) and blotted on 3 layers of autoclaved paper towels. The PYTHAR inoculum was prepared in diH_2_O and adjusted to a normalized OD_595_ of 1.5 (~10^6^ propagules per ml) as previously described [45]. Aqueous bacterial cell suspensions of S1Bt23 wild type (WT), Δ*phz*F, and Δ*phz*O mutants were prepared as previously reported by Chi et al. [12]. Double-folded autoclaved paper towels, moisturized with diH_2_O, were placed in a standard Petri dish (90 mm diameter), and 2 mL of the PYTHAR inoculum and bacterial cells were applied. The disinfected corn seeds were then randomly assigned to the following treatments: control (corn seed alone), PYTHAR alone, corn seed + bacteria, or corn seed + bacteria + PYTHAR. The treatments were arranged in a completely randomized design and incubated at 25 °C in triplicate, and diH_2_O was added to the autoclaved paper towels as required. Percentage seed germination and seedling vigor were collected 5 days after seeding. The vigor rating scale was as follows: 0, poor or dead; 1, moderate vigor (20–50% healthy roots); 2, high vigor (51–79% healthy roots); and 3, very high vigor (>80% healthy roots). Semi-quantitative scores were obtained as the product of percentage seed germination and seedling vigor, with a maximum score of 300 and a minimum score of 0. Statistical significance was calculated using one-way ANOVA and Fisher’s LSD Test performed using Graphpad Prism 6.0 software (GraphPad Software, Inc., San Diego, CA, USA).

### 2.3. Extraction of Secondary Metabolites: TLC and LC-MS Analyses

The protocol for the extraction of phenazines was adopted from Mehnaz et al. [46]. Strains of S1Bt23 were cultured in 100 mL of LB broth with shaking at 250 rpm for 90 h at 30 °C. Bacterial supernatants were separated from the pellet by centrifugation at 10,000 rpm for 10 min. Chloroform (15 mL) was added to 100 mL of bacterial supernatant, mixed thoroughly, and allowed to separate into layers. The upper (aqueous) phase was obtained and acidified with HCl to pH 3. Chloroform was then used to extract organic compounds from the aqueous phase, and the organic phase was allowed to evaporate to obtain solid residues. The crude extracts were re-dissolved in 1 mL methanol, transferred into 1.5 mL Eppendorf tubes, and stored briefly at 4 °C.

Pyrrolnitrin (PRN) extraction was performed from bacterial cell pellets as previously described by Souza and Raaijmakers [47] with modifications. Cells of S1Bt23 wild type and mutants were harvested from 15 mL cultures by centrifugation for 15 min at 10,000 rpm, and supernatants were discarded. The pelleted cells were then extracted twice with 5 mL ethyl acetate (99.9%; Sigma-Millipore, Oakville, ON, Canada) by shaking for 20–30 min at room temperature. The crude extracts were allowed to dry by evaporation in a fume hood at room temperature. The dried crude extracts were resuspended in 1 mL of methanol (99.8%; Sigma-Millipore) in 1.5 mL Eppendorf tubes and stored for 24 h at 4 °C.

TLC analysis of 20 μL extracts spotted on a 20 cm × 10 cm glass silica gel plate (Sigma, Canada) was performed as reported previously [45,47]. Synthetic phenazine-1-carboxylic acid (PCA) (Sigma, Canada), 2-hydroxyphenazine (2-OH-PHZ), and pyrrolnitrin (Ambeed, Arlington Heights, IL, USA) were dissolved in methanol and used as positive controls. The silica plate was placed into a glass chamber containing chloroform/acetic acid (49:1, *v*/*v*) as solvent (mobile phase). After about 2 h of sample migration, the silica plate was dried in the fume hood for 20 min at room temperature in the dark, followed by imaging on an ultraviolet (UV) transilluminator (Spectroline, Fisher Scientific, Ottawa, ON, Canada). For PRN, the TLC plates were sprayed with 2% p-dimethylaminobenzaldehyde in ethanol/sulfuric acid (1:1 *v*/*v*) (Ehrlich’s reagent, Sigma Aldrich, Oakville, ON, Canada) to detect purple spots (PRN).

LC-MS analysis of bacterial extracts was performed on the LTQ-Orbitrap XL coupled to the Dionex Ultimate 3000 UHPLC as previously described by Akuma et al. [48]. The chromatographic conditions of Brauer et al. [49] were used with a Kinetex column (C18, 2.1 × 50 mm, 1.7 µm) maintained at 30 °C and a flow rate of 0.350 mL/min as reported by Akuma et al. [48]. Molecular annotation was performed by comparison of exact *m*/*z* (±5 ppm), MS2 fragmentation pattern, and retention time to authentic synthetic standards of phenazine-1-carboxylic acid (PCA) and 2-hydroxyphenazine (2-OH-PHZ).

### 2.4. Phenazine-1-carboxylic Acid Toxicity Assay

Fresh overnight cultures of S1Bt23 were pelleted, and cells were resuspended in fresh Luria–Bertani (LB) broth. The optical density at 600 nm (OD_600_) was measured using a FLUOstar OPTIMA microplate reader (BMG LABTECH, Ortenberg, Germany) and standardized to an OD_595_ of 0.8. A 200-μL aliquot of the S1Bt23 cell suspension was placed in individual wells of a 96-well plate. Where required, the wells were amended with 50 mL of the corresponding stock solution to 250 μM or 500 μM PCA or an equal volume of methanol, in triplicate. Plates were sealed and incubated at 30 °C, and OD_600_ measurements were taken at 0 h, 24 h, and 48 h time points using the FLUOstar OPTIMA microplate reader. LB supplemented with an equal volume of methanol was used as a blank.

### 2.5. pAKanCRISPR–cas9 Deletion of phzO and prnC Genes

Generation of plasmids for CRISPR/Cas9 editing was performed as described previously for *phz*F deletion by our group [12]. The same protocol was used to design the single guide RNA (sgRNA) and other primers for CRISPR/Cas9-mediated deletions of *phz*O and *prn*C (Table 1). Preparation and DNA cloning of the single guide RNA (sgRNA) into the pAKanCRISPR vector to generate the pAKanCRISPR-sgRNA-HR was performed via the Gibson method as reported by Chi et al. [12]. For cloning purposes, the high-efficiency NEB^®^ 5-alpha competent *E. coli* cells (New England Lab, Woburn, MA, USA) were used for plasmid propagation and preparation. Sanger sequencing using the M13/pUC-R sequencing primer (Table 1) was used to confirm correct insertion and sequence identity.

The preparation of electrocompetent cells of *P. chlororaphis* subsp. *phenazini* S1Bt23 was performed as reported by Choi et al. [50]. A 50 μL aliquot of the electrocompetent cells was electroporated with parameters of 1.5 kV, 200 Ω, 25 μF, with 100 ng of pAKanCRISPR-sgRNA-HR plasmids using a Gene Pulser II Electroporator (BioRad, Mississauga, ON, Canada) as reported previously [12]. The electroporated cells were immediately amended with 1 mL of fresh LB medium and incubated for 1 h at 30 °C with shaking (250 rpm) to promote recovery. Colonies containing the pAKanCRISPR-sgRNA-HR plasmid were selected on LB agar plates supplemented with 50 μg/mL kanamycin. Colony PCR was performed using the specific HR1-F and M13/pUC-R primer set (Table 1) to confirm the presence of the pAKanCRISPR-sgRNA-HR plasmid. Positive colonies were selected and cultured in LB medium (10 mL) overnight.

For pCasPA transformation, S1Bt23 electrocompetent cells containing the pAKanCRISPR-sgRNA-HR plasmid were electroporated with 200 ng of pCasPA plasmid as described above [12,50]. One milliliter of fresh LB medium containing L-arabinose (2 mg/mL) was immediately added to the electroporated cells to induce the expression of the λ-Red system and Cas9 nuclease and incubated as indicated above for 2 h. LB agar containing 50 μg/mL kanamycin and 100 μg/mL tetracycline was used for *P. chlororaphis* subsp. *phenazini* S1Bt23 colonies containing pAKanCRISPR-sgRNA-HR and pCasPA. Successful deletion was confirmed by colony PCR amplification of the target gene using the corresponding screening primer sets (Table 1). Confirmed S1Bt23 mutants were then sub-cultured, cured, and preserved for downstream applications at  −80 °C in 15% glycerol, with the procedures performed as previously reported [12].

## 3. Results

### 3.1. PCA and 2-OH-PHZ Are the Major Phenazine Derivatives Produced by P. chlororaphis Subsp. phenazini S1Bt23

**Figure 1:**
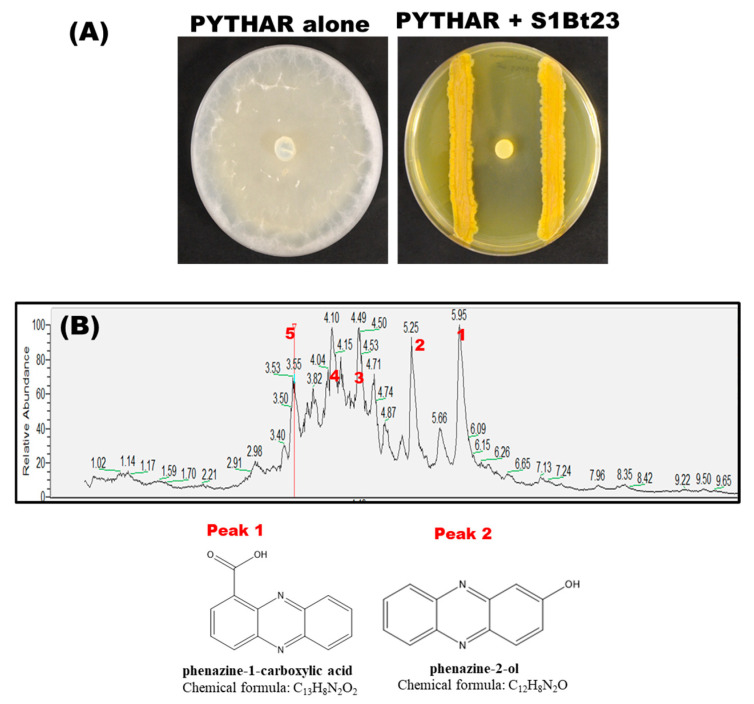
(**A**) Significant mycelial growth inhibition (95 ± 3%) of the oomycete *Pythium arrhenomanes* by *Pseudomonas chlororaphis* subsp. *phenazini* S1Bt23 (wild type) in dual culture assays: PYTHAR only (left) and PYTHAR+S1Bt23 (right). The assay was performed in glucose–casamino acid–yeast agar media with a mycelia-containing agar plug of PYTHAR inoculated in the center and S1Bt23 streaked equidistantly on either side. (**B**) Liquid chromatography–mass spectrometry (LC-MS) analysis of extracellular extracts from wild-type (WT) S1Bt23 extracts revealed the presence of phenazine-1-carboxylic acid (PCA; peak 1), 2-hydroxy-phenazine (2-OH-PHZ, peak 2), 3-benzylhexahydropyrrolo [1,2-a]pyrazine-1,4-dione (peak 3), 3-isobutylhexahydropyrrolo [1,2-a]pyrazine-1,4-dione (peak 4), and 3-isopropylhexahydropyrrolo [1,2-a]pyrazine-1,4-dione (peak 5). The chemical structure of the peaks of PCA and 2-OH-PHZ was deduced and validated using authentic synthetic standards.

We tested strain S1Bt23 against the maize root pathogen, *P. arrhenomanes*, in dual cultures using glucose–casamino acid–yeast agar (GCY*)*. S1Bt23 strongly inhibited the growth of *P. arrhenomanes* in in vitro cultures by 95 ± 3% (Figure 1A). We previously reported that *P. chlororaphis* subsp. *phenazini* S1Bt23 had potent antagonistic activity against the oomycete *Pythium ultimum* and produced phenazines [12], but the different phenazines were not characterized. We therefore sought to identify the different phenazines produced by *P. chlororaphis* subsp. *phenazini* S1Bt23. Liquid chromatography–mass spectrometry (LC-MS) analysis of the metabolic extracts of S1Bt23 (wild type) identified five prominent peaks (Figure 1B). Peaks 1 and 2 were the only detectable phenazine derivatives produced by *P. chlororaphis* subsp. *phenazini* S1Bt23 and were structurally identified as phenazine-1-carboxylic acid (PCA) and phenazine-2-ol (2-OH-PHZ) with predicted chemical formulae of C_13_H_8_N_2_O_2_ and C_12_H_8_N_2_O, respectively (Figure 1B). The remaining peaks were identified as 3-benzylhexahydropyrrolo [1,2-a]pyrazine-1,4-dione (peak 3), 3-isobutylhexahydropyrrolo [1,2-a]pyrazine-1,4-dione (peak 4), and 3-isopropylhexahydropyrrolo [1,2-a]pyrazine-1,4-dione (peak 5). The potential biosynthesis of phenazines was corroborated by whole-genome sequence (CP139026) analysis, which revealed an intact *phzABCDEFG* biosynthetic cluster in strain S1Bt23 (Figure S1). This cluster catalyzes the production of PCA from chorismate. Also, the *phz*O gene, which converts PCA into 2-OH-PCA before spontaneous decarboxylation to 2-OH-PHZ, was identified adjacent to the cluster. Hence, the whole-genome analysis of phenazine-related genes in S1Bt23 was consistent with the phenazine compounds detected in its extracts.

### 3.2. PCA Is Important for P. chlororaphis Subsp. phenazini S1Bt23 Antagonistic Activity Against P. arrhenomanes

**Figure 2:**
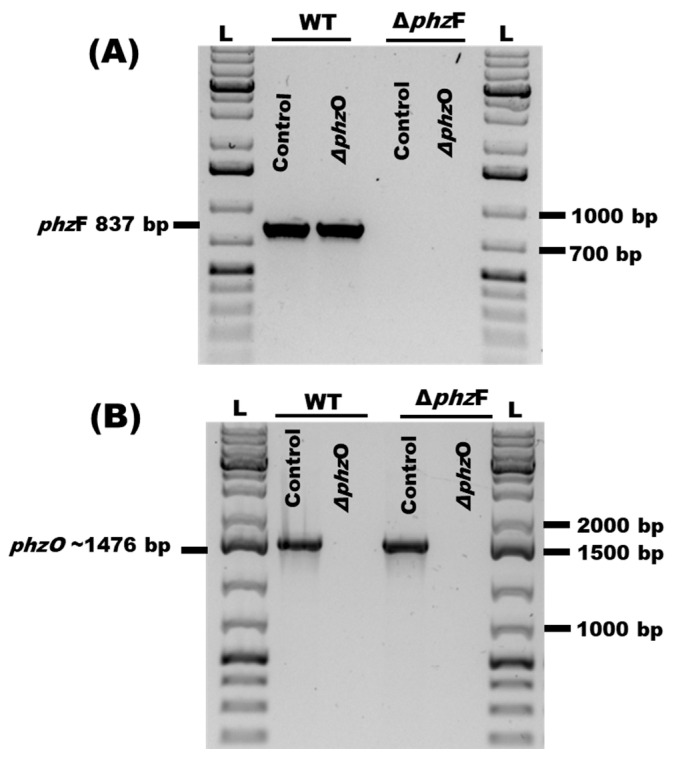
Confirmation of CRISPR/Cas9 knockouts of (**A**) *phz*F and (**B**) *phz*O genes by polymerase chain reaction amplification of genomic DNA samples extracted from the wild-type S1Bt23 (WT) and corresponding gene deletion mutants using specific primers. Expected sizes of ~837 bp and ~1476 bp for the *phz*F and *phz*O genes, respectively, were obtained. GeneRuler 1 kb bp Plus DNA Ladder (L) was used as a molecular weight marker.

To determine the relative contributions of PCA and 2-OH-PHZ to the potent antagonism of *P. arrhenomanes* isolate LEV1578 by *P. chlororaphis* subsp. *phenazini* S1Bt23, we performed CRISPR/Cas9 deletion of *phz*F, which is necessary for PCA synthesis, and *phz*O, which is necessary for 2-OH-PHZ biosynthesis (Figure S1). PCR amplification of *phz*F (Figure 2A) and *phz*O (Figure 2B) confirmed successful gene deletions in the corresponding mutant strains. For *phz*F gene deletion, the corresponding 837 bp was PCR amplified in wild type and Δ*phz*O but not in Δ*phz*F mutants (Figure 2A). Therefore, only the Δ*phz*O mutants did not show the required PCR amplicon of 1476 bp, which was present in the wild type and Δ*phz*F (Figure 2B).

**Figure 3:**
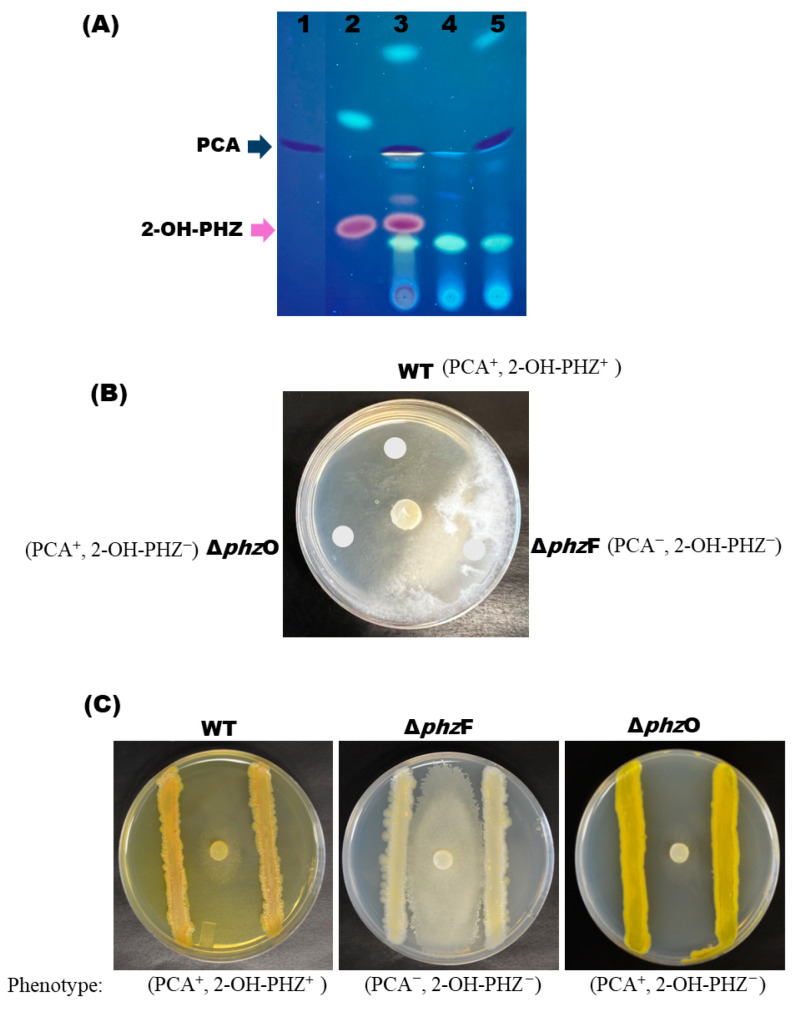
(**A**) Thin-layer chromatography (TLC) analysis of extracts from the supernatants of *Pseudomonas chlororaphis* subsp. *phenazini* S1Bt23 wild type (WT) and its Δ*phz*F and Δ*phz*O confirmed the absence of phenazine-1-carboxylic acid (PCA) and 2-hydroxy phenazine (2-OH-PHZ) in the respective mutants. Lanes 1, PCA synthetic standard; 2, 2-OH-PHZ synthetic standard; 3, S1Bt23 wild type; 4, Δ*phz*F mutant; and 5, Δ*phz*O mutant. Images of the TLC plates were obtained under UV light. (**B**) Bacterial extracts from WT and the D*phz*O mutant strongly inhibited the growth of *Pythium arrhenomanes* (PYTHAR), but the *Dphz*F mutant did not. Note the fluffy whitish mycelia where extracts of the DphzF mutant were spotted in glucose–casamino acid–yeast agar media with PYTHAR mycelia-containing plugs inoculated in the center. (**C**) The Δ*phz*F mutant lost the ability to inhibit the growth of *P. arrhenomanes* in dual culture assays, but WT and Δ*phz*O did not.

Thin-layer chromatography (TLC) analyses of the crude extracts were performed to determine whether PCA and 2-OH-PHZ production was successfully disrupted in Δ*phz*F and Δ*phz*O mutants (Figure 3A). TLC analysis with the authentic synthetic chemical standards revealed loss of PCA production in Δ*phz*F mutants only (blue arrow; Figure 3A) and loss of 2-OH-PHZ in both Δ*phz*F and Δ*phzO* strains (purple arrow; Figure 3A), which is expected as PCA is a precursor for 2-OH-PHZ synthesis (Figure S1). Liquid chromatography–mass spectrometry (LC-MS) analysis of extracts from S1Bt23 wild type and Δ*phz*F and Δ*phz*O mutants supported results from the TLC analysis (Figure S3). Interestingly, observations of wild type (WT), Δ*phz*F, and Δ*phz*O S1Bt23 cells on LB agar under white light revealed that deletion of either *phz*F or *phz*O is sufficient to cause loss of the characteristic orange pigment of *P. chlororaphis* subsp. *phenazini* S1Bt23 (Figure S2). Given that the common factor between Δ*phz*F and Δ*phz*O mutants is loss of 2-OH-PHZ synthesis (Figure S3), coupled with the fact that the pure 2-OH-PHZ standard has an orange pigment, we hypothesized that the characteristic orange pigment of S1Bt23 is directly linked to the production of 2-OH-PHZ.

Also, we performed dual culture assays to determine the relative contributions of PCA and 2-OH-PHZ to the biocontrol activity of *P. chlororaphis* subsp. *phenazini* S1Bt23 against *P. arrhenomanes*. The antagonistic activity of supernatant crude extracts (Figure 3A) and live cultures of *P. chlororaphis* subsp. *phenazini* S1Bt23 (wild type) and the Δ*phz*F and Δ*phz*O mutants against *P. arrhenomanes* was investigated. The crude extracts derived from the wild type (PCA^+^, 2-OH-PHZ^+^) and Δ*phz*O mutant (PCA^+^, 2-OH-PHZ^−^) significantly inhibited the mycelial growth of *P. arrhenomanes* (Figure 3B). In contrast, extracts from the Δ*phz*F mutant (PCA^−^, 2-OH-PHZ^−^) did not inhibit the mycelial growth of *P. arrhenomanes* (Figure 3B). Similarly, in dual cultures involving live cells, the deletion of *phz*F resulted in only 29 ± 3% radial mycelial antagonism of S1Bt23 against *P. arrhenomanes*. Δ*phz*O mutants had a 96 ± 3% inhibition, which is similar to the wild type and suggests no significant effect on the antagonistic activity (Figure 3C). The antifungal effects of live bacterial cells of wild type (PCA^+^, 2-OH-PHZ^+^) and Δ*phz*O mutant (PCA^+^, 2-OH-PHZ^−^) were both potent as expected (Figure 3C). These findings suggest that 2-OH-PHZ is not a major contributor to the antagonistic activity of S1Bt23 against *P. arrhenomanes*. As expected, the double knockout of *phz*F and *phz*O did not have a similar effect to the deletion of the *phz*F gene alone since *phz*O is downstream of *phz*F (Figure S1).

### 3.3. Corn Seed Bioprotection Assay by P. chlororaphis Subsp. phenazini S1Bt23

**Figure 4:**
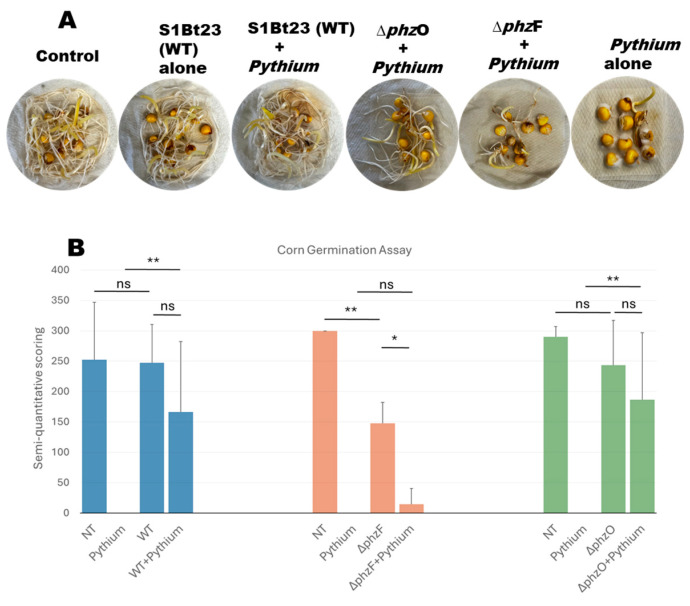
(**A**) Effective and significant bioprotection of corn seeds by *Pseudomonas chlororaphis* subsp. *phenazini* S1Bt23 against *Pythium arrhenomanes* pathogen in germination assays. Note: The vigor and germination rate in S1Bt23 + Pythium treatment compare favorably to those of the control and S1Bt23-alone treatments. (**B**) Semi-quantitative statistical analysis based on the product of the germination percentage of corn seeds and the seedling vigor ratings for each treatment. Vigor rating scale: 0, poor or dead; 1, moderate vigor; 2, high vigor; and 3, very high vigor, with a maximum score of 300 and a minimum score of 0. Statistical significance was calculated using one-way ANOVA and Fisher’s LSD Test. Results from three replicates are shown. * *p* < 0.05, ** *p* < 0.01, and ns, not significant. Error bars represent the standard deviation from the mean. NT, nontreated seeds.

A corn seed assay was also conducted to investigate whether S1Bt23 can bioprotect corn seeds against *P. arrhenomanes*. This study confirmed the aggressiveness of *P. arrhenomanes* isolate LEV1578 on corn inbred CO441, as 0–10% of the seeds germinated when treated with the pathogen alone. The germination rates (88.9%) and vigor of seedlings (3.0) of seeds treated with strain S1Bt23 alone were comparable and identical to nontreated control seeds (Figure 4A). Seeds in PYTHAR + Δ*phz*F treatments exhibited only a 37.0% germination rate (mean vigor 0.33), while those treated with PYTHAR + Δ*phz*O had 90% with a mean vigor of 2.0. A semi-quantitative scoring method (a product of percent germination and seedling vigor) was used to visualize the effects of the different treatments (Figure 4B). The lowest values were observed in treatments involving PYTHAR alone and PYTHAR + Δ*phz*F mutants (Figure 4B). The data from nontreated seeds and seeds treated with S1Bt23 (wild type) were not statistically different. Also, the nontreated seeds were not significantly different from seeds treated with Δ*phz*O mutants alone. However, seeds treated with Δ*phz*F mutants alone were statistically different from the nontreated corn seeds (Figure 4B). This demonstrated the potential involvement of PCA in the bioprotection of seeds against *P. arrhenomanes*, the root and stem rot pathogen of corn. Mutant S1Bt23Δ*phz*FΔ*phz*O with phenotypes indicated in Table 1 performed as Δ*phz*F mutants, with no antagonism against *P. arrhenomanes*, and as such, was not studied further.

### 3.4. PCA Is More Potent and Cell-Toxic than 2-OH-PHZ

We also demonstrated that more PCA is being produced compared to 2-OH-PHZ in the extracellular extracts of wild-type S1Bt23. Secondary metabolites, extracted using chloroform, air-dried, and resuspended in 1 mL of methanol, were quantified. LC-MS analysis of extracts of S1Bt23 (wild type) recorded average concentrations of ~3.74 mM and ~1.69 mM of PCA and 2-OH-PHZ, respectively. This suggests a production ratio of ~2.2:1, indicating that about 30% of the actual PCA produced is converted to 2-OH-PHZ by the *phz*O gene. Intriguingly, the average concentration of PCA produced in the Δ*phz*O mutant (PCA^+^, 2-OH-PHZ^−^) was 2.97 mM, which is lower than that of the wild type (3.74 mM). An indication that other repressor genes were triggered to regulate PCA biosynthesis, possibly due to the potential cell toxicity of the compound compared to 2-OH-PHZ.

**Figure 5:**
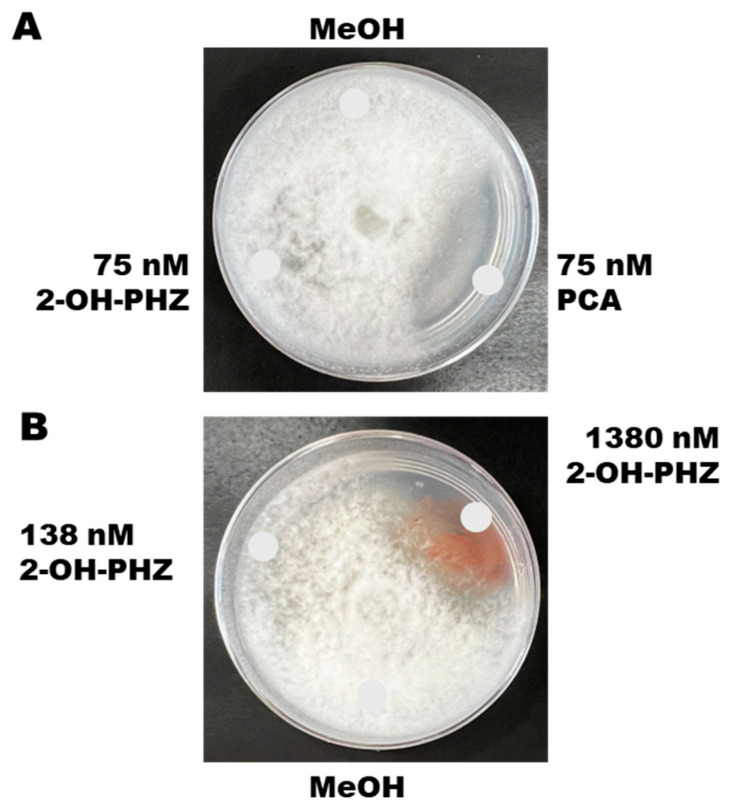
(**A**) Effects of synthetic phenazine-1-carboxylic acid (PCA) and 2-hydroxylphenazine (2-OH-PHZ) at 75 nM on mycelial growth of *Pythium arrhenomanes*. (**B**) Synthetic 2-OH-PHZ inhibited the growth of *P. arrhenomanes* only at very high concentrations of 1380 nM. MeOH, methanol used to dissolve the compounds, was included as a negative control. Glucose–casamino acid–yeast agar media were inoculated in the center with a plug of mycelia, and the chemicals were spotted at the indicated positions.

We tested the toxicity of synthetic compounds of the two phenazine derivatives against *P. arrhenomanes* on potato dextrose agar medium. Figure 5A shows the results of 75 nM solutions of PCA and 2-OH-PHZ on *P. arrhenomanes*. Synthetic PCA, at 75 nM concentration, strongly inhibited the growth of PYTHAR on PDA plates (Figure 5A). Interestingly, equimolar amounts of 75 nmol (Figure 5A) and 138 nM (Figure 5B) of 2-OH-PHZ remained insufficient to inhibit PYTHAR, suggesting that PCA is generally more potent and toxic than 2-OH-PHZ. However, it was only after a 10-fold increase in the concentration (1380 nM) of 2-OH-PHZ that we observed significant inhibition of PYTHAR (Figure 5B). As such, 2-OH-PHZ is less potent than PCA in suppressing mycelial growth of *P. arrhenomanes*. This suggests that 2-OH-PHZ might exhibit less toxicity to the cells of *P. chlororaphis* subsp. *phenazini* S1Bt23 compared to PCA.

**Figure 6:**
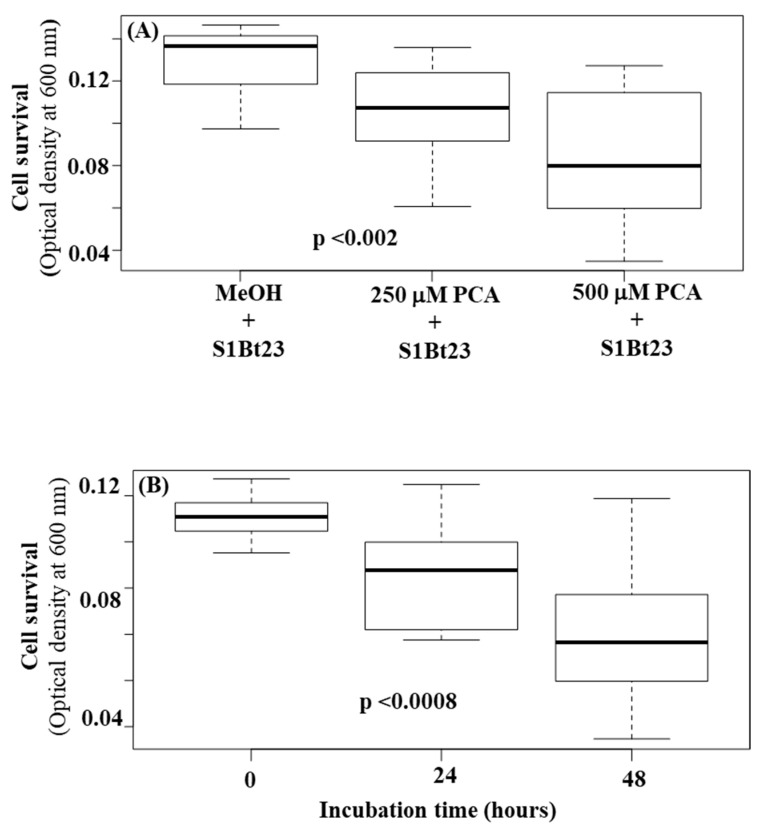
Boxplot visualization of the effect of phenazine-1-carboxylic acid concentration (**A**) and time of incubation (**B**) on the survival of *Pseudomonas chlororaphis* subsp. *phenazini* S1Bt23 cells incubated with methanol (MeOH; negative control). Blanked OD_600_ measurements were used. Statistical significance was calculated using two-way ANOVA followed by Tukey’s post hoc test. The probability values in each box denote significant differences between the three means.

We hypothesized that PCA could be toxic to bacterial cells since the deletion of the *phz*O gene did not lead to an increase in its biosynthesis, suggesting a potential tight cellular regulation. We grew S1Bt23 cells in culture medium amended with high concentrations (250 mM and 500 mM) of PCA, and their growth was monitored using optical density at 595 nm. Incubation with PCA resulted in significant cell death, with higher concentrations having a more pronounced effect (Figure 6A). At a concentration of 500 mM of PCA, the OD_6005_ dropped to a mean of 0.08 compared to ~0.13 in MeOH alone treatment, a 3-fold decrease (Figure 6A). As expected, the time of incubation negatively impacted the survival of *P. chlororaphis* subsp. *phenazini* S1Bt23 (Figure 6B). A strong, significant negative correlation (r = −0.994, *p* < 0.05) was observed between the optical density (cell growth) and PCA concentrations, suggesting decreased cell density (survival) when PCA concentration was increased from 250 mM to 500 mM. The interaction between incubation time and PCA concentration had statistically significant effects (*p* < 0.001). The viability of the cells was confirmed by plating aliquots of the treatments on LB agar medium. Hence, we showed that phenazines can be toxic even to a strain that produces PCA. This could explain why the biosynthesis of PCA can be strictly regulated as a protective mechanism against self-lethality. Details on the analysis of variance (ANOVA) are given in Table S1.

## 4. Discussion

Phenazines are a diverse group of compounds well known for their broad-spectrum antifungal and antibacterial properties [1,2,3,4]. As such, phenazines and the strains that produce them have been commercially adopted for the biocontrol of various phytopathogens, including oomycetes, fungi, and nematodes [51,52,53]. Phenazine derivatives tend to have distinct activities and toxicities owing to their diverse chemical properties. Hence, in strains that produce multiple phenazines, it is important to study the relative contributions of each phenazine derivative to their virulence and fitness as biocontrol agents.

Our group isolated strain S1Bt23 [44] and showed that it is a potent antagonist of eight key fungal and oomycete plant pathogens. Chi et al. [12] showed that phenazines are involved in its biocontrol of *P. ultimum* but did not investigate the effects of the different phenazine derivatives produced by *P. chlororaphis* subsp. *phenazini* S1Bt23. In the current study, we evaluated the effectiveness of *P. chlororaphis* subsp. *phenazini* S1Bt23 against *P. arrhenomanes*, as well as investigated the contribution of the two phenazine derivatives detected by TLC and LC-MS. TLC and LC-MS data confirmed that *P. chlororaphis* subsp. *phenazini* S1Bt23 biosynthesizes PCA and 2-OH-PHZ as its main phenazine compounds. This was corroborated by the presence of an intact *phz*ABCDEFG core cluster, responsible for PCA biosynthesis, in the genome of *P. chlororaphis* subsp. *phenazini* S1Bt23. In addition, adjacent to this core cluster, we identified the putative gene *phz*O (a flavin-dependent monooxygenase), required for the conversion of PCA to 2-hydroxyphenazine-1-carboxylic acid (2-OH-PCA) and quickly to 2-hydroxyphenazine (2-OH-PHZ). LC-MS analysis did not detect 2-OH-PCA in S1Bt23 supernatant extracts, suggesting that 2-OH-PCA is highly unstable and quickly converts to its more stable derivative, 2-OH-PHZ. Our study demonstrated that 30% of PCA may be converted into 2-OH-PHZ. CRISPR/Cas9 gene deletion of the gene *phz*F (Δ*phz*F) abrogated PCA production, and 2-OH-PHZ synthesis was lost with the deletion of the *phz*O (Δ*phz*O).

Using the generated *phz*F and *phz*O mutants, we were able to demonstrate that PCA is the major phenazine derivative involved in the biocontrol activity of S1Bt23 against *P. arrhenomanes*, the corn root and stem rot pathogen. The 2-OH-PHZ produced by *P. chlororaphis* subsp. *phenazini* S1Bt23 was not bioactive against *P. arrhenomanes*. While *phz*F deletion almost abrogated S1Bt23 antagonistic activity against *P. arrhenomanes*, deletion of *phz*O alone (no production of 2-OH-PHZ) had no effect. This suggests that PCA plays a significant role compared to 2-OH-PHZ in S1Bt23 antagonism against *P. arrhenomanes*. As expected, extracts from Δ*phz*O mutants could inhibit *P. arrhenomanes* *in vitro*, unlike extracts from Δ*phzF* mutants, likely owing to the presence of PCA in Δ*phzO* extracts. Chi et al. [12] reported that phenazines are involved in the antagonism of *P. ultimum* by the deletion of *phz*F, the gene that condenses the two identical trans-2,3-dihydro-3-hydroxyanthranilic acid (DDHA) molecules into the phenazine ring system for the synthesis of bioactive phenazine compounds [54]. The current study, using both Δ*phz*F and Δ*phz*O mutants, allowed for a better understanding of the key phenazine compound effective against *P. arrhenomanes*. This revealed that PCA, and not 2-OH-PHZ, is the key driver of the antagonism by *P. chlororaphis* subsp. *phenazini* S1Bt23.

To further demonstrate the potency of PCA in the biocontrol of *P. arrhenomanes* by *P. chlororaphis* subsp. *phenazini* S1Bt23, we tested the effectiveness of equimolar concentrations of synthetic PCA and 2-OH-PHZ. At low equimolar amounts of 75 nM, only PCA was able to significantly inhibit the growth of PYTHAR. Notably, 2-OH-PHZ was able to inhibit the growth of PYTHAR only at a significantly higher concentration of 1380 nM (18-fold higher than PCA), indicating that 2-OH-PHZ is much less potent than PCA against *P. arrhenomanes*. Our results are similar to those of Puopolo et al. [32], who found that the PCA compound exhibited antagonistic activity against phytopathogens tested, while 2-OH-PHZ showed low potency against fungal species. In contrast, a number of studies have shown that the antibacterial and antifungal activity of 2-OH-PHZ is stronger than that of PCA [13,35]. Hence, the activity of phenazine derivatives against different pathogens may be dependent on several factors, such as the relative concentrations of each metabolite, the producing strain, and the specific pathogen targeted.

Although a significant proportion of PCA is converted to 2-OH-PHZ by *P. chlororaphis* subsp. *phenazini* S1Bt23, 2-OH-PHZ did not appear to have much antagonistic activity against *P. arrhenomanes*. We therefore questioned the evolutionary benefit of this conversion. Phenazines have been described to have toxic effects by various mechanisms, including the induction of reactive oxygen species (ROS) and the destabilization of the electron transport chain [19,29,55,56,57,58]. We therefore hypothesized that although it is an important virulence factor, cellular overproduction of some phenazine derivatives, e.g., PCA, may be harmful to the producing bacterial cell, and as such, these compounds might be tightly regulated. Mirelles et al. [36] showed that PCA, pyocyanin, 1-hydroxyphenazine (1-OH-PHZ), and phenazine-1-carboxamide (PCN) produced by *P. aeruginosa* displayed toxicity towards the phenazine-deficient mutant. We postulated that 2-OH-PCA and PCA could be more toxic to the producing cell than 2-OH-PHZ due to the highly reactive radicals. For example, the 2-OH-PCA molecule has two key reactive radicals: -COOH and -OH. This could be why it is spontaneously converted to 2-OH-PHZ (less toxic) by the *phz*O gene. We demonstrated, using synthetic PCA and 2-OH-PHZ compounds, that the former is more toxic than the latter to the cells of S1Bt23 wild type. It is, therefore, a potential harm-reduction strategy for strain S1Bt23 to convert excess PCA to 2-OH-PHZ to limit cell toxicity. However, the deletion of *phz*O disrupted the modification of PCA to 2-OH-PHZ, but this did not promote additional PCA accumulation. This suggests that the regulation of PCA biosynthesis is tightly controlled as a protective mechanism, probably due to its potential high cell toxicity. As such, the functions of other genes may be triggered to normalize PCA biosynthesis. Other genes, such as *rpe*A, *rsm*E, and *lon,* have been reported to negatively regulate PCA biosynthesis in bacteria [59,60]. These data could have application in bioengineering the phenazine-producing ability of *P. chlororaphis* subsp. *phenazini* S1Bt23 to make it a more effective biopesticide.

The CRISPR/Cas9 knockouts of *phz*F and/or *phz*O demonstrated direct and significant involvement of phenazines in the antagonistic activity of S1Bt23. Based on the data, it can be concluded that the fungistatic and fungicidal effects are due to diffusible phenazines produced by *P. chlororaphis* subsp. *phenazini* S1Bt23. As such, we expected that the pathogen, *P. arrhenomanes,* would grow over the streaked cells of Δ*phz*F mutants. Intriguingly, the mycelial growth of *P. arrhenomanes* stopped at the edge of the streaked cells of Δ*phz*F mutants. This led us to hypothesize that poorly diffusible secondary metabolites are also produced by *P. chlororaphis* subsp. *phenazini* S1Bt23. Mining the whole-genome sequence of the strain S1Bt23 identified a complete biosynthetic gene cluster (*prn*ABCD; Figure S1) for the production of pyrrolnitrin [3-chloro-4-(2′-nitro-3′-chlorophenyl) pyrrole]. Pyrrolnitrin is produced by many *Pseudomonas* species and is reported to have broad-spectrum antifungal activity [61,62,63,64,65]. We demonstrated that the *prn*ABCD cluster is functional in strain S1Bt23 using gene expression studies and TLC analysis [48]. Due to the high hydrophobicity of pyrrolnitrin, it is highly probable that its diffusibility in aqueous environments such as agar is limited, making it more localized near and/or partly in the bacterial cell compared to more diffusible phenazine derivatives with polar groups. Given the strong previously reported antifungal effects of pyrrolnitrin, we postulated that it might contribute to the later restriction of mycelial growth of *P. arrhenomanes* in dual culture assays with streaked cells of Δ*phz*F mutants. This hypothesis was verified by the deletion of the *prn*C gene using CRISPR/Cas9 (Figure S4).

Single deletion of the *prn*C gene did not affect antagonistic activity of Δ*prn*C mutants, which was similar to S1Bt23 wild type, suggesting that pyrrolnitrin is not the major driver of the biocontrol activity of the strain against *P. arrhenomanes*. However, double knockout of the *phz*F and *prn*C genes completely abrogated the antagonistic activity of the derived mutants as the mycelia of the pathogen grew on and over the streaked cells of the Δ*phz*FD*prn*C mutant (Figure S4). Mutants S1Bt23Δ*phz*FΔ*prn*C and S1Bt23Δ*phz*FΔ*phz*O, with phenotypes indicated in Table 1, performed as Δ*phz*F mutants with no antagonism against *P. arrhenomanes*. This confirmed that PCA is the key driver of biocontrol activity of S1Bt23 and demonstrated some involvement of pyrrolnitrin later in the inhibition process of *P. arrhenomanes*. Huang et al. [59] reported that pyrrolnitrin played a more critical role in the suppression of *Fusarium graminearum* by *Pseudomonas chlororaphis* Go5 than phenazines. This is contrary to our results with *P. arrheromanes,* showing that PCA is the critical driver for *P. chlororaphis* subsp. *phenazini* S1Bt23. The specificity of the molecules could, thus, be pathogen-related. This is not surprising given that the cell walls of *Fusarium* (true fungus) are predominantly made of glycoproteins, chitin, β-1,3-glucan, and α-1,3-glucan [66,67], while *Pythium* (oomycetes, not true fungi) have cell walls rich in cellulose and β-1,3/β-1,6 glucans [68,69].

CRISPR-Cas9 technology has revolutionized bacterial genome engineering, and it is transforming biology [70]. The CRISPR-Cas9 system, originally discovered as an adaptive immune mechanism in bacteria and archaea, has become a powerful tool for precise genome editing in prokaryotic organisms. In bacteria, CRISPR-Cas9 enables targeted gene knockouts, insertions, and regulatory modifications, facilitating metabolic engineering, antibiotic resistance studies, and the development of synthetic biology applications [71,72]. One major advantage of CRISPR-Cas9 in bacterial systems is its high specificity and programmability [73]. Also, the CRISPR-Cas9 editing system is faster, more cost-effective, and more efficient than traditional methods based on λ-Red or transposon mutagenesis [74]. However, several key limitations exist, including the requirement for protospacer adjacent motif sequences that restrict target site selection, off-target cleavage that can lead to unintended mutations, and Cas9 expression toxicity that can hinder transformation efficiency in some bacterial strains [75]. Additionally, editing outcomes can be influenced since bacterial DNA repair mechanisms, particularly non-homologous end joining and homologous recombination, vary across species. Improvements are being engineered, such as base editors, CRISPR interference (CRISPRi), and Cas9 variants, to circumvent some of these issues while maintaining the versatility and precision of CRISPR-based bacterial genome manipulation [76,77,78].

## 5. Conclusions

In this study, we characterized the mechanism(s) involved in the potent antagonistic activity of *P. chlororaphis* subsp. *phenazini* S1Bt23 against *P. arrhenomanes*. CRISPR/Cas9 gene deletion studies revealed that phenazine-1-carboxylic acid is the key driver of the biocontrol activity of S1Bt23 against *P. arrhenomanes,* the root and stem rot pathogen of corn. We showed that ~30% of the PCA produced by *P. chlororaphis* subsp. *phenazini* S1Bt23 is converted to 2-OH-PHZ by the *phz*O gene. The deletion of the *phz*O gene, however, abrogated the 2-OH-PHZ biosynthesis but did not increase PCA production, suggesting that its production is tightly regulated by other repressors. This led to the conclusion that the strict PCA regulation could be an auto-protection mechanism by *P. chlororaphis* subsp. *phenazini* S1Bt23 cells against potential PCA toxicity. In addition, the study indicated that pyrrolnitrin plays a minor role in the antagonistic potency of *P. chlororaphis* subsp. *phenazini* S1Bt23. Finally, our study demonstrated that S1Bt23 can effectively bioprotect susceptible corn seeds against *P. arrhenomanes*, paving the way for greenhouse and field evaluations of *P. chlororaphis* subsp. *phenazini* S1Bt23 as a potential eco-friendly biofungicide and a valuable alternative to chemical fungicides.

## Figures and Tables

**Table 1:** Bacterial strains, plasmids, and oligonucleotide primers used in this study.

Strains/Plasmids/Primers	Characteristics, or Sequence 5′ → 3′ Orientation	Description	Source
**Bacterial strains** **:**			
*Pseudomonas chlororaphis:*			
S1Bt23	wild type, PCA^+^, 2-OH-PHZ^+^, PRN^+^, Car^r^, Strept^r^, Kan^s^, tet^s^	wild type, potent antagonist of *Pythium arrhenomanes*	Tchagang et al. [37]
S1Bt23Δ*phz*F	mutant, Δ*phz*F, PCA^−^, 2-OH-PHZ^−^	S1Bt23 derivative with *phz*F deleted	Chi et al. [12]
S1Bt23Δ*phz*O	mutant, Δ*phz*O, PCA^+^, 2-OH-PHZ^−^	S1Bt23 derivative with *phz*O deleted	This study
S1Bt23Δ*phz*FΔ*phz*O	mutant, Δ*phz*FΔ*phz*O, PCA^−^, 2-OH-PHZ^−^, PRN^+^	S1Bt23 derivative with *phz*F and phzO genes deleted	This study
S1Bt23Δ*prn*C	mutant, Δ*prn*C, PCA^+^, 2-OH-PHZ^+^, PRN^−^	S1Bt23 derivative with *prn*C gene deleted	This study
S1Bt23Δ*phz*FΔ*prn*C	mutant, Δ*phz*FΔ*phz*O, PCA^−^, 2-OH-PHZ^−^, PRN^−^	S1Bt23 derivative with *phz*F and *prn*C genes deleted	This study
*Escherichia coli*:	*E. coli* DH5alpha	Carrier of the plasmids	Addgene
**Plasmids:**			
CasPA	Tet^r^	Expression of Cas9 nuclease and λ-Red system	Addgene # 113347
pAKanCRISPR	pACRISPR plus Kan^r^, amp^s^	backbone for sgRNA expression with kanamycin resistance	Chi et al. [12]
**Oligonucleotides:**			
*phz*O CRISPR deletion:			
*phz*O g1-F	GTGGCAAGTCTTCTTTGTTTCTAG	*phz*O guide RNA 1 (sgRNA1)	This study
*phz*O g1-R	AAACCTAGAAACAAAGAAGACTTG		This study
*phz*O g2-F	GTGGCGTGTACCAATGGCTGTATG	*phz*O guide RNA 2 (sgRNA2)	This study
*phz*O g2-R	AAACCATACAGCCATTGGTACACG		This study
*phz*O HR1-F	gatctgtccatacccatggtCTAGATCGCCAGAGTGAAGAACTC	To amplify the left homology arm sequence of the *phz*O gene	This study
*phz*O HR1-R	gcaatcaggtGGTAGCAGCCTCAGTAATG		This study
*phz*O HR2-F	ggctgctaccACCTGATTGCCGTGTAGG	To amplify the right homology arm sequence of the *phz*O gene	This study
*phz*O HR2-R	tggcgggagtatgaaaagtcTCGAGGGTCTTGGGCTTTGGTATTG		This study
*phz*O KO screening F	GAGGCTGCTACCATGCTAGAT	To amplify *phz*O from the genomic DNA of colonies.	This study
*phz*O KO screening R	CGGCAATCAGGTCTATTTGGC		This study
prn*C* CRISPR deletion:			This study
*prn*C guide 1-F	GTGGTGAAGCGACACGGCTCTTCG	*prn*C guide RNA 1 (sgRNA1)	This study
*prn*C guide 1-R	AAACCGAAGAGCCGTGTCGCTTCA		This study
*prn*C guide 2-F	GTGGCGACGCCTATCTGTTGCAAG	*prn*C guide RNA 2 (sgRNA2)	This study
*prn*C guide 2-R	AAACCTTGCAACAGATAGGCGTCG		This study
*prn*C guide 3-F	GTGGAGTCGGTCACGCTCGTCTTC	*prn*C guide RNA 3 (sgRNA3)	This study
*prn*C guide 3-R	AAACGAAGACGAGCGTGACCGACT		This study
*prn*C HR1-F	gatctgtccatacccatggtCTAGAAACCGATCCGAGTCGGG	To amplify the left homology arm sequence of the *prn*C gene	This study
*prn*C HR1-R	tgtcgttcatGGGGGCAAACTCTCCTTG		This study
*prn*C HR2-F	gtttgcccccATGAACGACATTCAATTGGATCAAG	To amplify the right homology arm sequence of the *prn*C gene	This study
*prn*C HR2-R	tggcgggagtatgaaaagtcTCGAGGCGAAGTGCAGGTGCATG		This study
*prn*C KO screening F	GTACGAAGTCGGGCAAAGC	To amplify the *prn*C gene in colonies to confirm knockout (KO).	This study
*prn*C KO screening R	AGGGACGCTTCTTGATGCT		This study
M13/pUC-R	AGCGGATAACAATTTCACACAGG	Sequencing primer to confirm correct insertion of guide RNA and homology repair sequences into pACRISPR vector	Chi et al. [12]

Car^r^, carbenicillin resistant; Kan^r^, kanamycin resistant; Tet^r^, tetracycline resistant; Strept^r^, streptomycin resistant; amp^s^, ampicillin susceptible.

## Data Availability

The original contributions presented in this study are included in the article/Supplementary Materials. Further inquiries can be directed to the corresponding author.

## Supplementary Materials

The following supporting information can be downloaded at https://www.mdpi.com/article/10.3390/microorganisms14010019/s1, Table S1: Two-way ANOVA statistics for toxicity of phenazine-1-carboxylic acid; Figure S1: Complete phenazine (A) and pyrrolnitrin (B) biosynthetic clusters annotated in the genome sequence (CP139026) of *Pseudomonas chlororaphis* subsp. *phenazini* strain S1Bt23 using antiSMASH (Blin et al. 2023 [79]) and Prisma4 (Skinnider et al., 2020 [80]). Red stars indicate the genes deleted (*phz*F, *phz*O, and *prn*C) in this study; Figure S2: Colony phenotypes of S1Bt23 wild type (WT) and Δ*phz*F and Δ*phz*O S1Bt23 mutants on Luria–Bertani agar medium under white light. Note the loss of the characteristic orange color, which is related to the loss of 2-OH-PHZ; Figure S3: Liquid chromatography–mass spectrometry (LC-MS) analysis of extracellular extracts of the Δ*phz*O S1Bt23 mutant showed only a single peak corresponding to phenazine-1-carboxylic acid (PCA). As expected, 2-hydroxylphenazine (2-OH-PHZ) was not produced due to the deletion of the *phz*O gene. Synthetic PCA and 2-OH-PHZ were used as standards. Corresponding peaks are indicated; Figure S4: Minor additive effect of pyrrolnitrin (prn) produced by *Pseudomonas chlororaphis* subsp. *phenazini* S1Bt23 wild type (WT) against *Pythium arrhenomanes* is evident by CRISPR/Cas9 knockout of *prn*C and *phz*F genes.

## Abbreviations

The following abbreviations are used in this manuscript:

PYTHAR	*Pythium arrhenomanes*
LB	Luria–Bertini
PCA	Phenazine-1-carboxylic acid
2-OH-PHZ	2-Hydroxylphenazine
