# Supplementary material for: CRISPR/Cas9 Knockout Studies Implicate Phenazine-1-carboxylic Acid, but Not 2-Hydroxy Phenazine, in the Biocontrol Activity of Pseudomonas chlororaphis Subsp. phenazini Strain S1Bt23 Against Pythium arrhenomanes (Drechsler)
Source: Microorganisms. 2025 Dec 20;14(1):19. doi: 10.3390/microorganisms14010019 (PMC12844363; doi:10.3390/microorganisms14010019)
Supplement: Supplementary file 1 [file microorganisms-14-00019-s001.zip › microorganisms-3936247-supplementary.pdf]

Supplementary Table and figures

CRISPR/Cas9 Knockout Studies Implicate Phenazine-1-Carboxylic Acid Not 2-HydroxyPhenazine in Biocontrol Activity of *Pseudomonas chlororaphis* subsp. *phenazini* Strain S1Bt23 against *Pythium arrhenomanes*

Mercy Akuma, Sylvia Ighem Chi, Renlin Xu, Indira Thapa, Aida Kebede, Barbara Blackwell and James Tabi Tambong

Table S1

Figure S1

Figure S2

Figure S3

Figure S4

Table S1. Two-way ANOVA statisticsfor toxicity of phenazine-1-carboxylic acid

	Df	Sum Sq	Mean Sq	F value	Pr(>F)	
Incubation time	2	0.011368	0.00568	40.956	2.00e-07	***
PCA concentrations	2	0.010249	0.005124	36.926	4.26e-07	***
Interaction	4	0.002007	0.000502	3.615	0.0248	*
Residuals	18	0.002498	0.000139			

Signif. codes: 0 ‘***’ 0.001 ‘**’ 0.01 ‘*’ 0.05

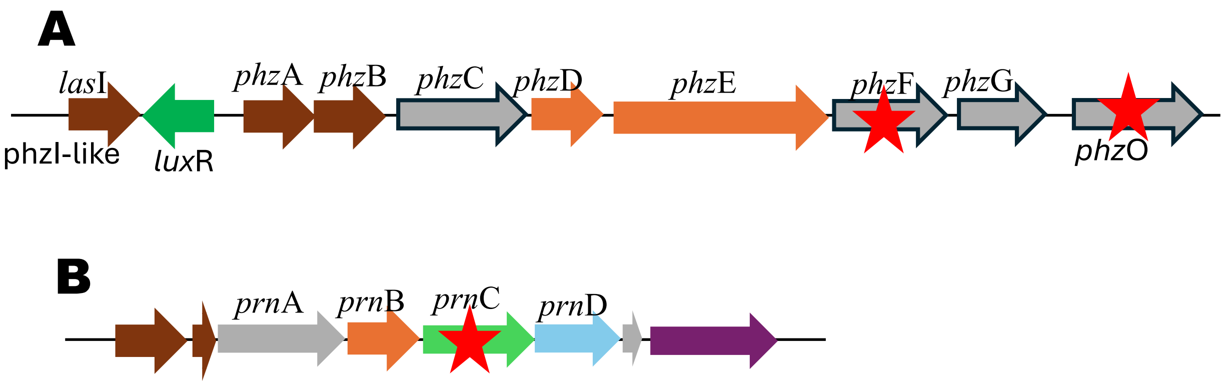

Figure S1. Complete phenazine (**A**) and pyrrolnitrin (**B**) biosynthetic clusters annotated in the genome sequence (CP139026) of *Pseudomonas chlororaphis* subsp. *phenazini* strain S1Bt23 using antiSMASH (Blin et al. 2023) and Prisma4 (Skinnider et al., 2020). Red stars indicate the genes deleted (*phz*F, *phz*O and *prn*C) in this study.

References

Blin, K.; Shaw, S.; Augustijn, H.E.; Reitz, Z.L.; Biermann, F.; Alanjary, M.; Fetter, A.; Terlouw, B.R.; Metcalf, W.W.; Helfrich, E. J.N; van Wezel, G.P.; Medema, M.H; & Weberanti, T. antiSMASH 7.0: new and improved predictions for detection, regulation, chemical structures, and visualization. Nucl Acids Res **2023**, doi: 10.1093/nar/gkad344.

Skinnider, M.A.; Johnston, C.W.; Gunabalasingam, M.; Merwin, N.J.; Kieliszek, A.M.; MacLellan, R.J.; Li, H.; Ranieri, M.R.M.; Webster, A.L.H.; Cao, M.P.T.; Pfeifle, A.; Spencer, N.; To, Q.H.; Wallace, D.P.; Dejong, C.A.; Magarvey, N.A. Comprehensive prediction of secondary metabolite structure and biological activity from microbial genome sequences. Nat Commun. **2020** Nov 27;11(1):6058. doi: 10.1038/s41467-020-19986-1.

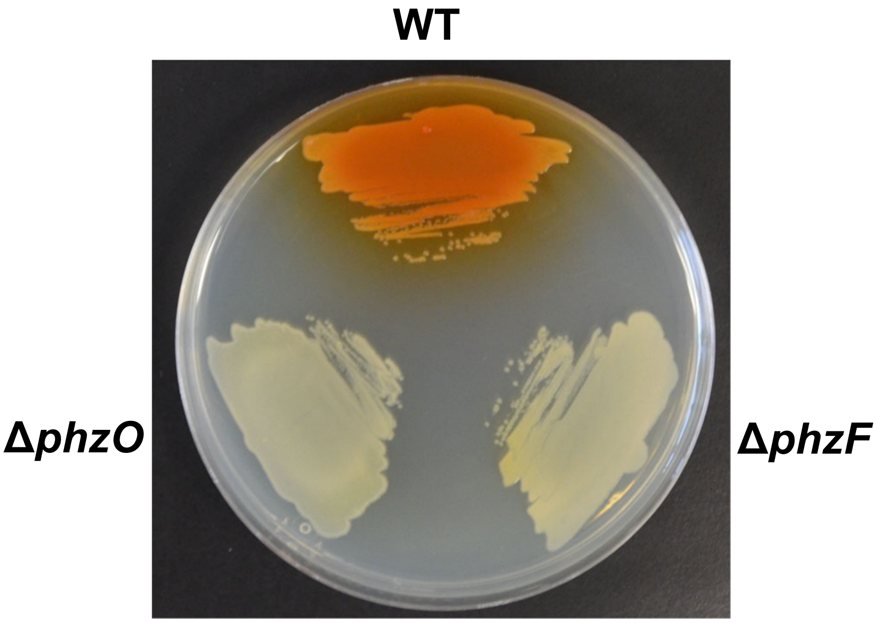

Figure S2. Colony phenotypes of S1Bt23 wild-type (WT), and Δ*phz*F and Δ*phz*O S1Bt23 mutants on Luria-Bertani agar medium under white light. Note the loss of the characteristic orange color which is related to the loss of 2-OH-PHZ.

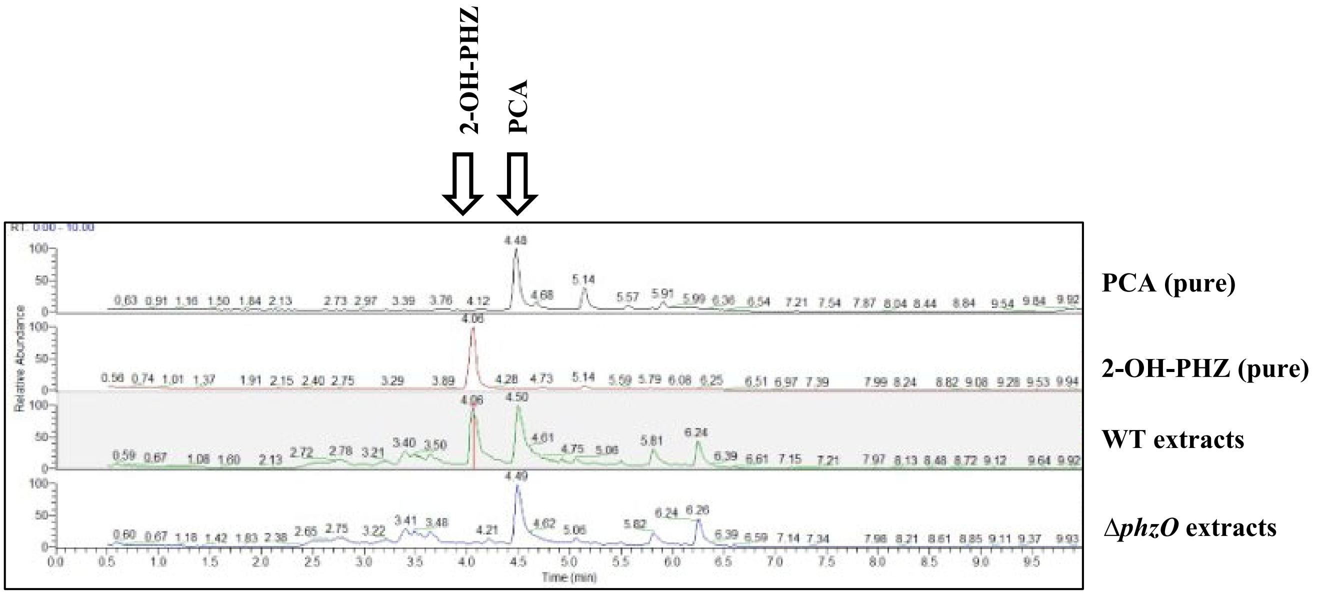

Figure S3. Liquid Chromatography Mass Spectrometric (LC-MS) analysis of extracellular extracts of Δ*phz*O S1Bt23 mutant showed only single peak corresponding to phenazine-1-carboxylic acid (PCA). As expected, 2-hydroxylphenazine (2-OH-PHZ) was not produced due to the deletion of *phz*O gene. Synthetic PCA and 2-OH-PHZ were used as standards. Corresponding peaks are indicated.

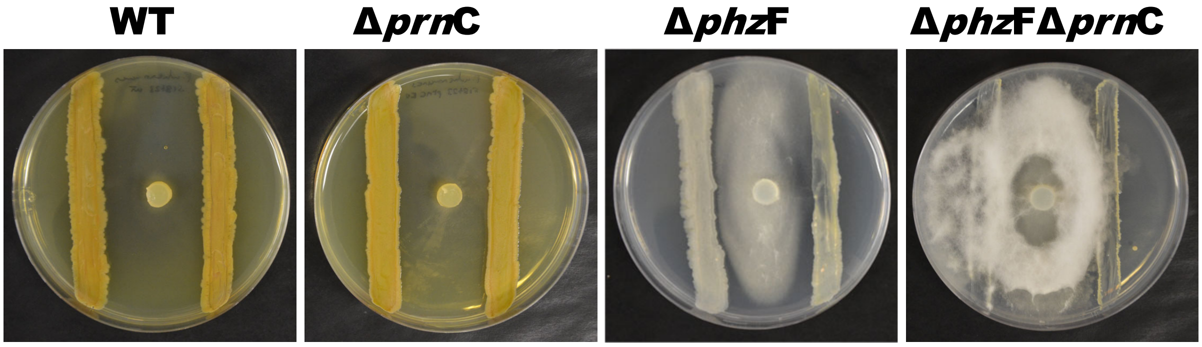

Phenotype: (PCA^+, 2-OH-PHZ^+, PRN^+) (PCA^+, 2-OH-PHZ^+, PRN^-) (PCA^-, 2-OH-PHZ^-, PRN^+) (PCA^-, 2-OH-PHZ^-, PRN^-)

Figure S4. Minor additive effect of pyrrolnitrin (prn) produced by *Pseudomonas chlororaphis* subsp. *phenazini* S1Bt23 wild-type (WT) against *Pythium arrhenomanes* is evident by CRISPR/Cas9 knockout of *prn*C and *phz*F genes. Δ*prn*C is mutant with *prn*C deleted while in Δ*phz*F mutant, the *phz*F gene is deleted. Note: double deletion of *prn*C and *phz*F (Δ*phz*FΔ*prn*C) genes shows *Pythium* growing over the streaked bacteria. Antagonistic activity of S1Bt23 is mainly due to PCA production.
